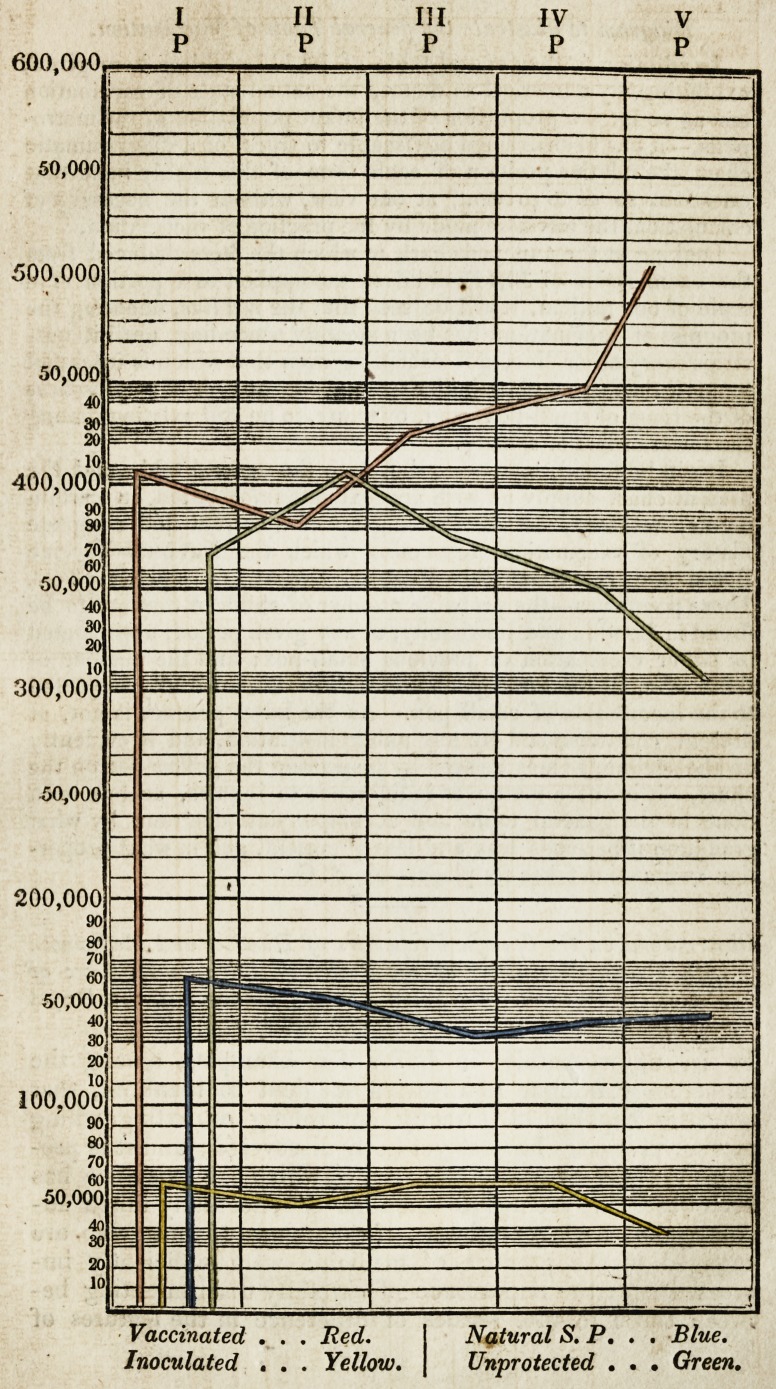# Documents Exhibiting the Actual State of Vaccination among 30, 117 Children of the Poor in the Metropolis; Drawn up from Observations and Calculations Made at the Royal Metropolitan Infirmary for Sick Children

**Published:** 1826-08

**Authors:** A. B. Granville


### COMMUNICATIONS.

*Documents exhibiting the actual State of Vaccination among* 30,117 *Children of the Poor in the Metropolis; drawn up from Observations and Calculations made at the Royal Metropolitan Infirmary for Sick Children.* By A. B. GRANVILLE, M.D. F.R.S.

AT this season of increased alarm at the prevalence of small-pox in the metropolis and various parts of the country, it cannot be an act of presumption to suppose that every thing which relates to the inoculation of cow-pox will be received with interest by the profession.

The subject of vaccination has very recently engaged, in an unusual degree, the attention of the public and of government. This has principally arisen from the vague assertions that have been made respecting it, inducing the better-informed classes of society to entertain wavering opinions respecting cow-pox, notwithstanding the able and conclusive Report from the National Vaccine Board, the members of which, and its indefatigable president, took such pains to investigate the question.

The efforts made by those individuals have placed the truth in so clear a light, that any further observation may be deemed unnecessary. But, as its demonstration can be further promoted by a collated mass of well-digested facts, found in the registers of the Royal Metropolitan Infirmary for Sick Children, I have arranged and computed a general table of the state of vaccination among the children admitted during the last five years and a half, in which every point connected with their history, as far as it relates to small-pox and cow-pox, is faithfully recorded: supplying, thus, positive, instead of only presumptive, evidence of the incorrectness (and in many instances the groundlessness) of the reports lately disseminated respecting the pretended inefficacy of vaccine inoculation.

The general table to which I allude, and which the directors and governors of the Institution have deemed of sufficient importance to be printed and circulated among the subscribers, may, perhaps, be considered as not unworthy of a place in the New Series of the London Medical and Physical Journal; in which case, a few explanatory observations, and an illustrative diagrammatic chart, will enable the reader to comprehend more readily that document, rich in facts highly favourable to a system from which parents have derived the greatest measure of comfort.

The materials out of which the general table is constructed are supplied by the parents of the children, when the latter are registered on their admission. On such an occasion, and touching such an object, the parents can have no possible motive for deceiving the medical officers; so that the information, thus obtained, may be presumed to be accurate. They are asked whether the child, whose age has been previously ascertained, has had the small-pox natural or inoculated; whether it has been vaccinated, or remains yet unprotected. Whenever small-pox has occurred after vaccination, the parents have been found ready enough to supply that fact, which, after as accurate an investigation into the reality of the case as can be instituted, is regularly registered. The whole of this information is arranged in appropriate columns, under the superintendence of the respective physicians and surgeons; and a monthly report of it is made by them to the directing committee. The facts thus learned extend, at this moment, to upwards of 30,000 children, under the age of thirteen years, whose history, in regard to small-pox and vaccination, has thus been narrowly inquired into.

From these facts the following conclusions are derived:

1st. That the practice of vaccination is steadily gaining ground among the poorer classes of society.

2d. That the inoculation of small-pox is of rarer occurrence than heretofore.

3d. That in proportion as the former practice increases, and the latter decreases, so have the cases of natural small-pox diminished.

4th. That parents, in general, are much less indifferent than hitherto to the great advantage of securing their offspring, by an early vaccination, from the ravages of small-pox.

5th. That small-pox after vaccination has been of very rare occurrence; and, when it has occurred, it has been mild in its course, and harmless.

6th and lastly. That, although two cases of death, referred to small-pox after vaccination by the parent, have been reported to the medical officers since the first foundation of the Infirmary, positive proofs do not exist of the real nature of those cases ; and they have, therefore, not been noticed in the table.

The computations contained in the general table have been made in two ways. In the first, the proportions are given, as deduced from the actual numbers registered, in units and fractions. In the second, the proportions are set down in decimal fractions only, deduced from what the numbers registered would have given, had they extended to a million: these latter calculations being still founded on the real numbers. In pursuing the second mode of calculating, I had the satisfaction of being assisted by my friend Mr. Finlaison, the able actuary of the National Debt Office.

Those who, to the language of numbers, prefer that which speaks to the eye, will probably find the diagrammatic chart, with which I have accompanied the present communication, more useful. Here inspection alone will show that the *red line,* denoting the progress of vaccination, rises higher in the fourth and fifth period (1824-1825), because at that time small-pox, after having sensibly diminished during the preceding year, had (as may be seen by the *blue line,*) again increased, and raged almost epidemically; so that more parents flew to the resource of cow-pox, in hopes of saving their children. This latter circumstance caused a corresponding and a remarkable depression in the *green line,* intended to mark the diminution of prejudice against, or carelessness respecting, cow-pox.

With regard to the *yellow line* of the chart, which is meant to exhibit the extent to which the inoculation of small-pox has been carried within the whole period embraced by that document, it speaks for itself. This baneful practice, which, more than any prejudice against vaccination, opposes itself to the full and glorious result of the Jennerian discovery, by keeping up the *fomes* of contagion intended to be extinguished by the cow-pox, is fortunately on the decline, and must continue to decline, if the members of the medical profession act with firmness. With what regret, then, must the public witness any attempt to perpetuate, by the proposition of extravagant experiments, a loathsome disorder, which, as the experience of many continental districts has proved, may be completely eradicated, after having existed in Europe for a period of thirteen centuries, by a steady, persevering, and, I may add, correct performance of vaccination!

16, *Grafton-street, Berkeley-square ;*
*7th of June,* 1826.

**A GENERAL TABLE,** embracing the Medical History of Thirty Thousand One Hundred and Seventeen Children, (under Thirteen Years of Age,)

*As far as the same relates to the occurrence of Natural Small-Pox, or to the Inoculation of Small-Pox and Cow-Pox; showing the* progressive increase of the latter Practice, *and corresponding Decrease of Inoculation, with the effect of both on Smalt-Pox generally.*

| *Periods.* | Number of Children admitted. P. | S. | | Of whom had been Vaccinated. P. | S. | | Proportion of the Vaccinated to the Admitted. | Supposing the Admitted to have been 1,000,000, then the Vaccinated would be | Had been Inoculated with the Small-Pox. P. | S. | | Proportion of the Inoculated to the Admitted. | Supposing the Admitted to have been 1,000,000, then the Inoculated would be | Had had the natural Small Pox. P. | S. | | Proportion of the Natural Small-Pox to the Admitted. | Supposing the Admitted to have been 1,000,000, then those who have had the Nat. Sm. P. would be | Had been left unprotected. P. | S. | | Proportion of the Unprotected to the Admitted. | Supposing the Admitted to have been 1,000,000, then the unprotected would be | Proportion of the Unprotected to the Vaccinated. | Number of Cases of Small-Pox after Vaccination. | Proportion of such Cases among the Vaccinated. |
|---|---|---|---|---|---|---|---|---|---|---|---|---|---|---|---|---|---|---|---|---|---|---|---|---|---|---|
| *1st Period.* Oct. Nov. Dec. 1820. 1821. Jan. Feb. Mar. 1822. | 6053 | 2422 | 8475 | 2461 | 995 | 3456 | One in 2.45 | 407,787 | 324 | 200 | 524 | One in 16.17 | 61,829 | 947 | 413 | 1360 | One in 6.23 | 160,479 | 2321 | 814 | 3135 | One in 2.70 | 369,911 | One in 1.10 | 6 | One in 575 |
| *2d Period.* From 1st April, 1822 To 1st April, 1823. | 4738 | 2291 | 7029 | 1791 | 910 | 2701 | One in 2.60 | 384,265 | 244 | 127 | 371 | One in 18.95 | 52,781 | 680 | 400 | 1080 | One in 6.50 | 153,649 | 2023 | 854 | 2877 | One in 2.44 | 409,304 | One in 0.93 | 4 | One in 675 |
| *3d Period.* From 1st April, 1823 To 1st April, 1824. | 3239 | 1957 | 5196 | 1361 | 848 | 2209 | One in 2.30 | 425,135 | 148 | 170 | 318 | One in 16.34 | 61,201 | 433 | 266 | 699 | One in 7.43 | 134,527 | 1297 | 673 | 1970 | One in 2.63 | 379,138 | One in 1.17 | None | None |
| *4th Period.* From 1st April, 1824 To 1st April, 1825. | 3265 | 1711 | 4976 | 1434 | 796 | 2230 | One in 2.23 | 448,151 | 187 | 114 | 301 | One in 16.53 | 60,490 | 438 | 262 | 700 | One in 7.70 | 140,675 | 1206 | 539 | 1745 | One in 2.85 | 350,683 | One in 1.27 | 2 | One in 1115 |
| *5th Period.* From 1st April, 1825 To 1st April, 1826. | 3000 | 1441 | 4441 | 1469 | 786 | 2255 | One in 1.53 | 507,768 | 83 | 87 | 170 | One in 26.12 | 38,280 | 459 | 185 | 644 | One in 6.89 | 145,012 | 989 | 383 | 1372 | One in 3.24 | 308,939 | One in 1.65 | 11 | One in 205 |
| Totals . . . . . | 20,295 | 9822 | | 8516 | 4335 | | Average Proport. One in 2.4 | Average Proportion 426,702 | 986 | 698 | | Average Proport. One in 17.8 | Average Proportion 55,915 | 2957 | 1526 | | Average Proport. One in 6.8 | Average Proportion 148,853 | 7836 | 3263 | | Average Proport. One in 2.7 | Average Proportion 368,529 | Average Proport. One in 1.16 | 23 | Average Proport. One in 559 |
| General Totals . . | | | 30,117 | | | 12,851 | | | | | 1684 | | | | | 4483 | | | | | 11,099 | | | | None severe | |

30,117

OBSERVATIONS.—This inquiry extends to a period of seventeen years and a half, being two-thirds of the time since vaccination was first practised. The children being admitted until the age of twelve years, all those who were examined and registered in 1821, represent, as far as they are concerned, the state of the practice of vaccination for the space of twelve years previous to 1821; to which are to be added, the children admitted in the course of the last five years and a half. It is also to be remarked, that the 30,117 children, examined as to the question of small-pox and cow-pox, equal one-fourth of the total number of births that have taken place during the last five years and a half, within the Bills of Mortality. So that we have the means, by this Table, of making up our mind as to the real merit of vaccination, as far as that practice extends to one-fourth of the births in the metropolis, and for a period equal to two-thirds of the time since that practice was first adopted.

Looking at this Table as affording important data for calculating the progress of vaccination, it appears that, out of a mass of 100,000 children, taken indiscriminately from among the poorer classes of the population of the metropolis,

42,670 will be found to have been vaccinated,
36,852 ——— to be still unprotected,
14,885 ——— to have had the natural small-pox,
5,591 ——— to have been inoculated, and
179 only to have undergone, during this period, an attack (generally modified) of small-pox, notwithstanding previous vaccination; a proportion so small, that blind, indeed, must that individual be to the blessings of vaccination, who can consider so trifling an exception as a proof of the failure of the Jennerian discovery.

*Vaccinated . . . Red.* | *Natural S. P. . . Blue.*

*Inoculated . . . Yellow.* | *Unprotected . . . Green.*

*Diagram to illustrate the general Table of Vaccination.*

In addition to the general table of 30,117 children here given, exhibiting, by numerical evidence, the actual state of vaccination among so large a proportion of the infant population of the metropolis,—it has been deemed adviseable to trace, on a diagrammatic chart, the relative position of each term of this highly important question, so as to present, at one view, without the necessity of calculation, the advance made by the practice of vaccination.

Looking at the annexed chart, in which the facts deduced from the examination of 30,117 children are applied to a portion of a scale of one million, it will be seen that the *red line*, denoting the progress of vaccination, has been steadily ascending, until it outstrips every other line, not excepting even that which is intended to mark the degree of prejudice against cow-pox, or of carelessness of the issue of small-pox, said, by many, to be still existing among the lower orders of society.

It will be found, moreover, that both the general table and the present chart supply us with approximate information on certain points, necessary for the drawing-up of a correct and complete history of vaccination, respecting which the National Vaccine Board and the Small-Pox Hospital Report are equally silent. Those points are—the probable number of children that are to be found in London and its vicinity, at any given period, unprotected by either vaccination or previous small-pox; and the number of those who, in the same given period, have been wilfully subjected to the inoculation of small-pox. As the latter practice is not, at present, countenanced by any public institution, and is evidently on the decline, as may be seen by inspecting the *yellow line* on the chart,—it is not a matter of indifference to inquire, as has been done in the general table and accompanying diagram, by what gradation it becomes less and less prevalent, and in what proportion vaccination takes its place.—A. B. G.

## Figures and Tables

**Figure f1:**